# Bone Marrow Lymphoid Niche Adaptation to Mature B Cell Neoplasms

**DOI:** 10.3389/fimmu.2021.784691

**Published:** 2021-12-08

**Authors:** Erwan Dumontet, Stéphane J. C. Mancini, Karin Tarte

**Affiliations:** ^1^ Univ Rennes, Institut National de la Santé et de la Recherche Médicale (INSERM), Établissement Français du Sang (EFS) Bretagne, Unité Mixte de Recherche (UMR) U1236, Rennes, France; ^2^ CHU Rennes, Pôle de Biologie, Rennes, France

**Keywords:** B-cell non-Hodgkin lymphomas, cancer-associated fibroblasts, extracellular vesicles, tumor microenvironment, stroma cell

## Abstract

B-cell non-Hodgkin lymphoma (B-NHL) evolution and treatment are complicated by a high prevalence of relapses primarily due to the ability of malignant B cells to interact with tumor-supportive lymph node (LN) and bone marrow (BM) microenvironments. In particular, progressive alterations of BM stromal cells sustain the survival, proliferation, and drug resistance of tumor B cells during diffuse large B-cell lymphoma (DLBCL), follicular lymphoma (FL), and chronic lymphocytic leukemia (CLL). The current review describes how the crosstalk between BM stromal cells and lymphoma tumor cells triggers the establishment of the tumor supportive niche. DLBCL, FL, and CLL display distinct patterns of BM involvement, but in each case tumor-infiltrating stromal cells, corresponding to cancer-associated fibroblasts, exhibit specific phenotypic and functional features promoting the recruitment, adhesion, and survival of tumor cells. Tumor cell-derived extracellular vesicles have been recently proposed as playing a central role in triggering initial induction of tumor-supportive niches, notably within the BM. Finally, the disruption of the BM stroma reprogramming emerges as a promising therapeutic option in B-cell lymphomas. Targeting the crosstalk between BM stromal cells and malignant B cells, either through the inhibition of stroma-derived B-cell growth factors or through the mobilization of clonal B cells outside their supportive BM niche, should in particular be further evaluated as a way to avoid relapses by abrogating resistance niches.

## Introduction

B-cell non-Hodgkin lymphomas (B-NHL) are a heterogeneous group of hematological malignancies that emerge from different stages of normal mature B-cell differentiation (1). Lymphoma evolution and treatment are complicated by a high prevalence of relapses (2) primarily due to the ability of malignant B cells to interact with protective lymph node (LN) and bone marrow (BM) microenvironments (3–5). In agreement, several studies have correlated BM involvement with worsened prognosis and impaired chemotherapeutic response in B-cell lymphomas (6–8). This review delves into the current knowledge of the BM stromal cell modifications induced by the protumoral niche establishment in B-NHL with a specific focus on diffuse large B-cell lymphoma (DLBCL), follicular lymphoma (FL), and chronic lymphocytic leukemia (CLL). Interestingly, these three B-NHL subtypes displayed various BM involvement, with 11%-34% of DLBCL (9, 10), 70%-80% of FL (11), and virtually all CLL cases showing BM infiltration at diagnosis (**Table 1**). Moreover, this review highlights the newly described role of extracellular vesicles (EVs) in the seeding of the BM niche. EVs are released during homeostasis and cell activation, with pleiotropic effects on signaling between cells. EV cargos are enriched in nucleic acids, proteins, and lipids. Briefly, the International Society of Extracellular Vesicles had classified EVs into three main groups: i) exosomes, the small vesicles with diameters ≤100-150 nm that are formed inside multivesicular bodies; ii) microvesicles, medium-size vesicles of plasma membrane origin with diameters of up to 1000 nm; and iii) apoptotic bodies, the large vesicles with diameters > 1000 nm that are produced by apoptotic cells (12). Excellent reviews on the biomolecular and functional characteristics of EVs as well as on the techniques used for EV isolation and characterization have recently been published (13, 14).

**Table 1:** Key elements involved in the generation of B-cell non-Hodgkin lymphomas bone marrow supportive niches.

	DLBCL	FL	CLL
**BM involvement** (% of cases)	11-34%	70-80%	All
**Pattern**	Mixed: from localized focal infiltrates to complete disruption	Nodular aggregates admixed with lymphoid-like TME	Mixed nodular-interstitial, interstitial, and diffuse
**BM stroma factors involved in B-cell homing**	Unknown	CXCL12	CXCL12 and VLA-4
		CCL19, CXCL13	
**BM stroma factors involved in B-cell survival**	BAFF	Hehghog ligands	BAFF, CD44,
	IL-6 IL-17A	BAFF, TGF-β, VLA-4,	Plexin-B1, CXCL12, C1q
		CXCL12	
**Metabolic reprogramming induced by BM TME**	Unknown.	BM B cells are metabolically less active than LN B cells	BM stromal cells release glutathione and trigger CLL glycolytic shift
**Effects of tumor EVs on BM stromal cells**	Unknown.	↗CXCL12, ↗ ANGPT1, ↗ KITLG, ↗IL-7	↗VEGF
			Inflammatory pro-tumoral phenotype

DLBCL is the most common aggressive B-NHL and accounts for approximatively 24% of new NHL cases (15). Gene expression analysis and study of genomic alterations have identified distinct genetic subtypes in DLBCL, reflecting differential pathogenesis, and associated with distinct clinical behavior (16–19). Interestingly, recent studies have highlighted the impact of tumor microenvironment (TME) heterogeneity on tumor B-cell biological features and on DLBCL patient outcome (20, 21).

FL accounts for about 20% of adult lymphoma and is an indolent disease characterized by prolonged periods of remissions preceding relapses and ultimate transformation into DLBCL in about 30% of cases. The genetic hallmark of FL is the t (14, 18) translocation occurring during the V(D)J recombination of immunoglobulin genes in the BM. The resulting deregulation of BCL2 provides a selective survival advantage to B cells during the germinal center (GC) reaction, triggering illegitimate recirculation of t (14, 18)^pos^ post-GC B cells detectable in most healthy individuals. Iterative (re)entry of these FL precursor cells inside GC favors accumulation of additional genetic alterations sometimes converging towards overt FL (22). Importantly, FL is the paradigm of a neoplasia fully dependent on a complex microenvironment network that coevolves with tumor B cells to create a tumor supportive niche in both LN and BM (23, 24).

CLL is the most common hematologic malignancy in adults in Western countries. CLL is preceded by a stage of monoclonal B-cell lymphocytosis and is characterized by the accumulation of mature clonal B cells resistant to apoptosis in the blood, BM, and lymphoid organs. Patients with CLL have a heterogeneous clinical course with some never needing treatment, while others require treatment immediately after diagnosis or during illness due to a more symptomatic and unfavorable clinical course. In typical CLL cases, the tumor B cell clone exhibits an abnormal expression of markers like CD5, CXCR4, and ZAP-70, that are used to stratify the disease in conjunction with the mutational status of the BCR reflecting different cell of origin (25, 26). Despite fully disseminated presentation, TME provides crucial survival signals to malignant CLL cells within the proliferation centers of LN and BM (27).

In these three mature B-cell neoplasms, specialized tumor niches support survival, proliferation, and drug resistance of tumor B cells. These highly heterogeneous niches include defective tumor immunity, due to altered recruitment and cell exhaustion of cytotoxic cells, to the amplification of immunosuppressive cells, or to immune escape mechanisms developed by tumor B cell themselves, hampering tumor recognition, immune synapse formation, or anti-tumor cell activation (23, 24, 27). Conversely, fully functional tumor permissive cells, including CD4^pos^ T cell, myeloid cell, and stromal cell subsets, could be found. The relationship between LN and BM protumoral niches and how the similarities and differences between these microenvironments could impact malignant B-cell features remains elusive. In FL, malignant B cells found in the BM are characterized by a lower cytological grade, a decreased proliferation, and a reduced CD10 expression compared with LN FL B cells (28). Moreover, their gene expression profile reflects their reduced proliferation and active metabolism (29). Finally, somatic hypermutation analysis and targeted deep sequencing demonstrate that different FL B-cell subclones could be detected within LN *versus* BM, and suggested that FL originates in the LN and infiltrates BM early in the course of the disease, allowing further accumulation of BM-specific mutations (28, 30, 31). Besides the exact cell composition and supportive signals provided by BM niches, a major issue remains to establish how these niches evolve during tumor development, from the pre-tumoral stage to overt lymphoma, during remissions and relapses.

## Lymphoma BM Stromal Microenvironment

BM constitutes the primary site for the maintenance and differentiation of hematopoietic stem cells (HSCs) and for B-cell lymphopoiesis. Different stromal cell niches dynamically control these processes. Seminal papers have recently proposed a molecular atlas of the BM stromal cells at the single cell resolution, including osteoblasts, perivascular cells, endothelial cells, and mesenchymal stromal cells, providing clues on how various stromal cell subtypes could interact with HSCs and differentiating B-cell subsets (32–34). In the context of B-NHL, dynamic interactions between BM stromal cells and tumor B cells have been described to play a key role in converting the BM TME into a tumor supportive niche (34–36). DLBCL, FL, and CLL display distinct patterns of BM infiltration (**Table 1**). DLBCL show a mixed pattern of BM involvement that can potentially range from localized focal infiltrates to complete disruption of BM by lymphoma cell proliferation (37). In contrast, FL infiltration is primarily localized to the paratrabecular regions as nodular aggregates admixed with lymphoid-like TME (38). In CLL several BM infiltration patterns can be found including mixed nodular-interstitial, interstitial, and diffuse (39). In each cases, stromal cells exhibiting specific functional phenotype support recruitment, survival, and proliferation of tumor B cells, mimicking the cancer-associated fibroblasts (CAFs) described in solid cancers.

### BM Stromal Cells Support B-Cell Recruitment

BM DLBCL-CAFs have been poorly explored *in situ*. In contrast, in FL, BM-CAFs, like their LN counterparts, overexpress CXCL12 involved in the recruitment, adhesion, and activation of FL B cells (40) (**Table 1**). Moreover, they ectopically express CXCL13 and CCL19, the two lymphoid chemokines classically expressed by LN follicular dendritic cells (FDC) and fibroblastic reticular cells (FRC) respectively, thus recreating GC-like structures able to recruit and support CXCR5^pos^CCR7^pos^ FL B cells (41, 42).

CLL B lymphocytes could be attracted *in vitro* to BM stromal cells whose protective effects require close cell proximity (43–45). This colocalization of CLL tumor cells with their supportive stromal cell niche relies on the deregulation of several chemokine pathways (**Table 1**). The demonstration that the clinical efficacy of BCR inhibitors in CLL is mediated, at least in part, by the inhibition of chemokine receptor activity and the corresponding mobilization of tumor cells out of their protective niches further highlights the crucial role of stromal cell-derived chemokine in CLL survival (46). First, high expression of CXCR4 on the surface of peripheral blood CLL cells triggers their migration to BM stromal cells producing CXCL12 (45, 47–49). CXCR4 surface expression is regulated by its ligand, thus explaining the decrease in CXCR4 expression on tissue tumor B cells, while recirculating CLL B cells express high levels of CXCR4. In parallel, blood CLL cells express high amounts of CCR7 (50). Indeed, the recycling of CXCR4 and CCR7 receptors is potentiated in CLL cells and contributes to their stronger expression (51). Recently, it was shown that p66Shc (SHC-transforming protein 1), which limits the recycling of CXCR4 and CCR7 by inhibiting their de-phosphorylation, is deficient in CLL (52). Interestingly, CCR7 could also form heterodimers with CXCR4 thus disrupting the CXCR4/CXCL12 downstream signaling and reducing B-cell retention within BM (53). Furthermore, other proteins expressed by CLL cells, such as ZAP70 or CXCR7 have been shown to regulate the function of CXCR4 (54, 55). Altogether, the modulation of CXCR4 function could regulate the homing capacity of CLL cells within BM. Second, CXCR5, the CXCL13 receptor, is also expressed at high levels by CLL cells (56, 57). However, conversely to the ectopic induction of CXCL13-expressing FDC in FL BM, CXCL13 seems to be only involved in CLL B cell homing into LN and the increase of CXCL13 level in the plasma of CLL patients is correlated with LN size but not BM infiltration (58). Finally, integrin α4β1 (VLA-4) plays a prominent role in the homing of CLL cells to BM niches. VLA-4 major ligands, fibronectin and VCAM-1, are constitutively present on BM stromal cells and endothelial cells and are upregulated by inflammatory signals in a NF-kB-dependent manner (59). In mouse xenograft models, CLL cells from VLA-4^neg^ patients showed significantly lower BM homing rates than those from VLA-4^pos^ patients. In contrast, the spleen homing rates did not significantly differ. Clinically, the VLA-4 status directly drives in the extent of human BM infiltration (60).

### BM Stromal Cells Support B-Cell Survival

In DLBCL, the upregulation of Notch-3 in tumor cells under close cell-cell contact with BM-derived stromal cells has been implicated in the development of aggressive lymphoma cells (61). In turn, such direct interaction between DLBCL cells and stromal cells mediates an increase in B-cell activating factor (BAFF) expression by stromal thus resulting in a decrease of chemotherapy-induced B-cell apoptosis (62, 63) (**Table 1**). One of the factors involved in the regulation of DLBCL B-cell interaction with the BM stromal niche is the level of Jun expression. Indeed, Jun-regulated genes mediate the interaction of malignant cells with stromal cells and extracellular matrix proteins and impact extranodal localization (64). There is also evidence for tumor permissive effects of BM stromal cells on DLBCL cells through secretion of IL-6 and IL-17A, which promote both cell proliferation and drug resistance (8). Finally, the crosstalk between malignant B cells and stromal cells in DLBCL could also impact metabolic reprogramming in DLBCL. DLBCL have been early considered as metabolically heterogeneous (65, 66). Non-malignant cells from TME including stromal cells have been proposed to contribute to DLBCL metabolism by providing metabolic intermediates (67) but no data specifically address this issue in BM versus LN niches even if the use of specific metabolic inhibitors have been recently explored in some DLBCL subsets (68).

In FL, tumor B cells are strongly dependent on direct interactions with a microenvironment close to that of normal GC, including in particular follicular helper T cells (Tfh), myeloid cells, and lymphoid stromal cell subsets (23, 24, 69). The protumoral role of infiltrating lymphoid stromal cells has been demonstrated in particular by the identification of ectopically-induced FRC- and FDC-like cells within invaded BM (40, 70). To date the origin and heterogeneity of the stromal cells supporting FL B cells within LN and BM are not perfectly understood and it is very likely that several FL CAF subtypes co-exist and organize different cell niches with specific functions (38). Stromal cells supporting FL B cell survival have been initially identified as lymphoid-like stromal cells obtained *in vitro* by stimulation of BM mesenchymal precursors by TNF-α (TNF) and Lymphotoxin-α1β2 (LT) or by direct contact with malignant B cells (3). Interestingly, BM stromal cells obtained from FL patients display a specific gene expression profile even after *in vitro* amplification, suggesting an imprinting on these cells by the tumor context (40, 63, 71). VLA-4, which is expressed by FL-CAFs, is involved in the growth of GC lymphomas and their resistance to anti-CD20 treatments (72). *In vitro*, FL stromal cells decrease tumor B cell apoptosis through a set of partially resolved mechanisms, including the production of hedgehog ligands (Hh), BAFF and TGF-β, over- expression of ABC-type multi-drug transporters, and activation of a c-MYC/HDAC6 loop in tumor cells (24, 73). Moreover, CXCL12 contributes to FL B cell activation and synergize with BCR signaling (40). To date, the metabolism of FL remains broadly unexplored. Gene expression profile of FL B cells obtained from medullary niche reveals a decreased expression of the genes involved in of glycolysis, fatty acid synthesis, and OxPhos pathway compared to LN B cells (29). However, the role of stromal cells from BM versus LN niches in FL B-cell metabolic reprogramming remains to be evaluated.

CLL B cells could interact with stromal cells *via* different receptor/ligand couples including ICAM-1/LFA-1 (74), VCAM-1/VLA-4 (75–78), CXCR5/CXCL13 (79), BCMA/BAFF, or TACI/BAFF (80), or by transpresentation of IL-15 from stromal cells to B cells (81). Amon those, ICAM-1, VCAM-1 and BAFF have been shown to be expressed by BM stromal cells. These interactions could lead to leukemic cell survival *via* a CD44-dependent mechanism involving up-regulation of MCL-1 in CLL B cells (82), activation of NF-κB pathway (80), and result in migration and proliferation of leukemic cells. In the same way, the interaction between CD100 (on CLL B-cell surface) and Plexin-B1 (present on BM stromal cells) extends CLL B cell viability and enhances proliferation (83). The mutual activation of stromal cells and tumor cells also depends on the CLL-mediated activation of Notch2 in BM stromal cells, leading to C1q overexpression the reciprocal activation of the canonical Wnt pathway in CLL cells (84) Moreover, BM stromal cell derived CXCL12 exhibits a pro-survival effect on CLL tumor cells (44, 85, 86). BM Stromal cells may also induce protective epigenetic modifications in CLL B cells including hypomethylation of the lysine 27 of histone H3 protein subunit (H3K27me3) (87). Finally, BM stromal cells have an important role on CLL metabolism. CLL cells have a net increase of reactive oxygen species (ROS) compared to their normal counterpart and are highly sensitive to cellular antioxydants, such as glutathione, to maintain their redox balance. BM stromal cells trigger glutathione synthesis by CLL cells through cysteine release, thus protecting tumor cells from drug-induced apoptosis (88). Moreover, BM stromal cells contribute to the glycolytic shift in CLL cells, at least in part by the Notch/Myc axis, triggering an increased glycolysis associated with higher lactic acid production, glucose uptake, and glucose transportation (89, 90).

### BM Stromal Cells Organize the Tumor Niche

Beyond these functions of direct B-cell support, lymphoma CAFs are thought to be the organizers of the tumor niche. A role for the composition of the stromal-cell derived extracellular matrix in the pathogenesis of DLBCL was recently identified within tumor LN, raising the question of its direct and indirect impact on tumor growth, as an example through the modulation of immune cell infiltration, within invaded BM (21).

FL-CAFs overexpress the chemokine CCL2 within invaded BM, thus triggering the recruitment of monocytes that are then converted into pro-angiogenic and anti-inflammatory macrophages (71). FL tumor-associated macrophages have been shown to play a key role in the growth of FL B cells through the transpresentation of IL-15 and the triggering of BCR-dependent signaling involving DC-SIGN-expressing macrophages and oligomannose residues introduced in FL BCR (91, 92). BM and LN FL-CAFs could also promote the recruitment and survival of pro-tumoral neutrophils through the release of large amounts of IL-8 (63). Of note, in DLBCL, tumor cells have been shown to produce themselves IL-8 involved in the recruitment of APRIL-producing neutrophils (93). Moreover, BM and LN FL-infiltrating stromal cells also overexpress the immunosuppressive molecule PGE2 (94) involved in the recruitment or activation of suppressor cells such as Tregs and MDSCs (95). Finally, CAFs have been shown in solid tumors to physically hamper the recruitment of cytotoxic T cells to the tumor and CD8^pos^ T cells are retained at the periphery of FL tumor aggregates in both LN and BM, suggesting that FL-CAFs could contribute to tumor exclusion in lymphomas (96–98).

Overall, it is clear that close interactions of tumor B cells with stromal cells within the BM, together with modulation of chemokines and cytokines directly influence the growth of DLBCL, FL and CLL, providing evidence that the BM niche plays a critical role in both lymphoma survival and drug resistance. Regardless of their cell of origin, the mechanisms underlying the differentiation of lymphoma CAFs are of the utmost importance given their potential as therapeutic targets.

## Emergence of the BM Lymphoma Stromal Microenvironment

FL tumor B cells could directly contribute to the commitment of BM stromal precursors into an FRC-like phenotype overexpressing CCL2 and IL-8 through TNF-dependent mechanisms (3, 63, 71). Moreover, even if they produce less LT than normal centrocytes, the large number of GC-like B cells ectopically found in invaded FL BM probably contributes to a local overproduction of LT that synergizes with TNF for the induction of lymphoid stroma commitment. However, surrounding non-malignant cells could also participate in the polarization of FL-CAFs. Neutrophils, recruited by IL-8-producing BM FL stromal cells, could in turn contribute to their differentiation into FRC-like cells through activation of the NFκB pathway (63). In addition, LN FL-Tfh overexpress IL-4 which induces a Transglutaminase^hi^Podoplanin^low^CD106^hi^CXCL12^hi^ phenotype on human stromal cell precursors. FL-Tfh also produce high amounts of TNF and LT, which sensitize stromal cell precursors to the effect of IL-4, notably through increased expression of the STAT6 signaling molecule (40). Even if fully mature Tfh have not been detected within FL BM, IL-4 and CXCL12 have been shown to be correlated in invaded FL BM (40). Finally, some of the recurrent genetic alterations in FL regulate the re-education of the tumor niche by tumor B cells. In particular, the gain-of-function mutations of the histone methyltransferase EZH2, which occurred early in 20% to 30% of FL, are proposed to uncouple GC B cells from the critical Tfh checkpoint whereas switching them to FDC dependency (99). EZH2-mutated GC B cells downregulate many genes linked to Tfh signaling, fail to engage Tfh, thus limiting recycling toward the dark zone of GC, and survive in the light zone as proliferating centrocytes overexpressing LT, TNF, and BAFFR, all involved in GC B-cell/FDC crosstalk. HVEM loss-of-function mutations detected in about 40% of patients with FL have been associated, in a murine model of FL and in FL patients, with an amplification of Tfh producing large amounts of IL4, TNF, and LT, and able to activate FL-CAF within LN (100). No study had currently evaluated how these genetic events could impact FL TME co-evolution within BM. Even if such data are essentially lacking in the context of DLBCL, some recurrent genetic alterations have been recently associated with a specific TME pattern, with some of them related to overexpression of genes associated with GC-like stroma or extracellular matrix/FRC/CAF genes (21).

Finally, LT produced by CLL cells is involved in the polarization and/or *in situ* generation of the tumor stromal network and the secretion of CXCL13, IL-6, and IL-8 (74, 79). Moreover, the leukemic clone produces retinoic acid in the stromal microenvironment which contributes, at least in part, to the CXCL13 induction (101).

In addition to the factors described above, tumor derived EVs seem to be involved in the communication between tumor cells and their TME, in particular CAFs. Such mechanism could play a central role in triggering initial induction of tumor-supportive niche within distant sites, including BM.

## Roles of EVs in the Induction of BM Lymphoma Stromal Niche

**Figure 1:**
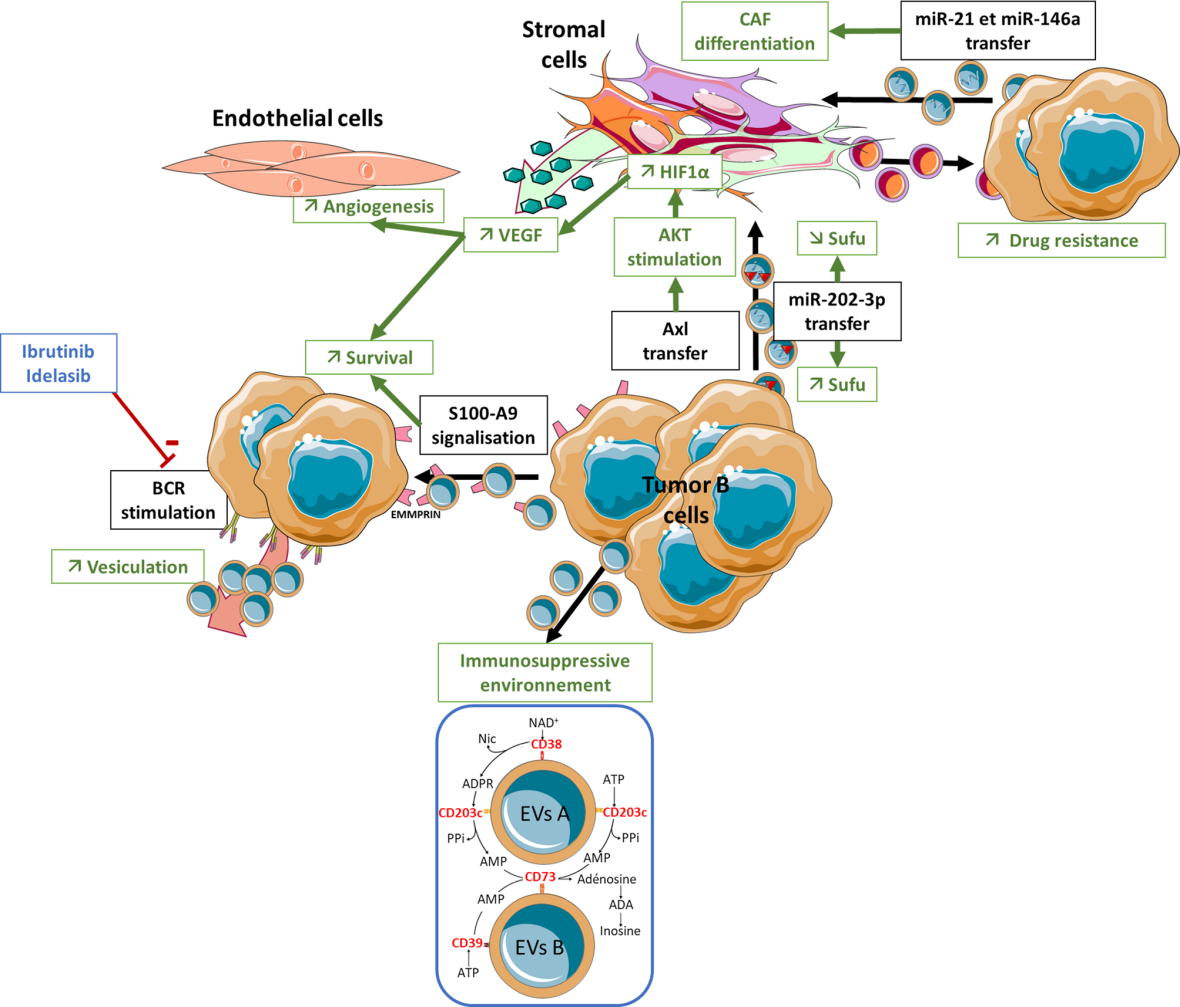
Role of EVs in the lymphoma microenvironment structuration. A bidirectional crosstalk has been reported between CLL and FL tumor B-cells and stromal cells *via* EVs that can give rise to a highly organized pro-tumor niche. ANGPT, Angiopoietin; BCR, B Cell Receptor; BM, Bone Marrow; CAF, Cancer Associated Fibroblast; EVs, Extracellular Vesicles; TGF, Transforming Growth Factor-β; VEGF, Vascular Endothelial Growth Factor.

To date no study has explored the putative involvement of EVs in the induction of a BM lymphoma stromal niche in the context of DLBCL. Moreover, only few studies have investigated the involvement of EVs in the pathophysiology of FL (**Figure 1**). Recently, FL-derived EVs were shown to modulate the gene expression profile of BM stromal cells, triggering an upregulation of HSC niche factors including CXCL12, angiopoetin-1, KITLG, or IL-7, and increasing the capacity of stromal cells to interact specifically with BM FL B cells and support their survival and their quiescent phenotype (29). Interestingly, the phenotype of EV-treated stromal cells is quite different from that obtained under treatment by TNF/LT or coculture with FL B cells supporting a role of EVs in the activation of BM stromal cells before BM seeding by malignant B cells. In fact, the level of CXCL12 is increased in non-involved BM plasma suggesting that FL EVs could shape the BM stromal niche before BM infiltration by tumor cells or at distance from this BM infiltration (unpublished data). In the same way, the analysis of the gene expression profile of BM stromal cells highlights a *continuum* ranging from healthy donor BM stromal cells, to stromal cells obtained from FL patients without BM involvement, and finally from FL-invaded BM (29). Altogether these data suggest that EVs could contribute to CXCL12 upregulation in the absence of direct contact with malignant B cells and could then synergize with IL-4 produced by infiltrating T cells admixed with FL B cells to further enhance local CXCL12 production. Interestingly, BM stromal cells activation by FL-derived EVs was shown to rely on TGF-β dependent pathways something that is reminiscent of the role of TGF-β in the B-cell/stromal cell crosstalk within FL LN (42). How TGF-β and STAT6 pathways could synergize for the acquisition of FL CAF phenotype within FL BM remains to be explored.

Bidirectional crosstalk has also been reported between CLL B-cells and their surrounding stroma *via* EVs (**Figure 1**). CLL B cells release large amounts of exosomes that show strong expression of CD37, CD9, and CD63. Ibrutinib, a Btk inhibitor, significantly reduces the amount of plasma exosomes in CLL patients. Likewise, *in vitro* treatment of CLL cells with Idelalisib (a PI3K inhibitor) decreases exosome secretion, something that is not observed during treatment with fludarabine (102). This result highlights the role of the BCR-PI3K pathway in controlling exosome secretion in CLL. Besides BCR itself, CLL supportive TME produces BAFF, APRIL, CD31, and plexin B1 that all protect CLL cells from spontaneous apoptosis by synergizing with BCR signaling (44, 103) and could influence EV secretion. The comparison of the mRNA content of EVs produced by B cells from healthy donors *versus* patients with CLL, and stimulated or not through the TLR9 pathway, shows enrichment for the kinases of the BCR pathway, LYN, SYK, MAPK1, MAPK2, and the anti-apoptotic proteins BCL2 and BCL3 in CLL-derived EVs. These EVs released by tumor B cells transfer their mRNA content to non-malignant cells in the TME (104). Microvesicles derived from malignant CLL cells and detected in peripheral blood also deliver the receptor tyrosine kinase Axl into BM stromal cells leading to the activation of a AKT/mTOR/p70S6K/HIF-1α axis resulting in an increase in VEGF synthesis (105). This increase in VEGF is associated with an increased neovascularization in medullary (106) and extramedullary tissues, as well as a paracrine pro-survival stimulation of tumor B cells (107). The miRNA content of CLL B cell-derived exosomes is strongly enriched in miR-21, miR-155, miR-146a, miR-148a, and let-7g (108). BM stromal cells treated *in vitro* with these CLL exosomes acquire an inflammatory pro-tumoral phenotype, while endothelial cells increase their capacity for angiogenesis (108). These effects are consistent with what is known about the effect of miR-21 and miR-146a in the transition from normal fibroblast to CAFs (109–112). Indeed, CLL miR-146a^pos^ exosomes induce the transition of BM stromal precursors into CAFs showing over-expression of α-SMA and FAP (113). In addition, CLL exosomes show specific enrichment in miR-202-3p, able to decrease expression of Sufu (a component of the hedgehog pathway) in stromal cells and to trigger stromal cell proliferation (114). Finally, EVs isolated from cultures of CLL BM stromal cells induce a significant decrease in spontaneous apoptosis of tumor B cells and an increase in their chemoresistance to several drugs, including fludarabine, ibrutinib, idelalisib, and venetoclax. In addition, these EVs induce changes in the gene expression profile of CLL cells mimicking the transcriptomic signatures obtained after BCR stimulation (115).

## Disrupting the EV “Remote Communication” to Improve Lymphoma Prognosis

Analyzing the deregulation of extracellular proteins or miRNAs in the blood and tumor niches of patients during B cell tumorigenesis is a reliable tool for the identification of new tumor-targeted therapies. For example, the detailed mode of action of the CD30 antibody-drug conjugate Brentuximab vedotin in DLBCL is not well understood since the clinical outcome seems to be partially independent of the CD30 expression on the tumor cells. However, as CD30^pos^ bystander cells are enriched in the tumor tissue in many cases of DLBCL, CD30 might be released within TME-derived EVs. Thus a model was proposed in which even in the absence of CD30 on the tumor cells, EVs can transport the targeting protein from cells of the TME to tumor cells (116). This model would explain the clinical efficacy of Brentuximab vedotin also in cases of lack of the targeting antigen on tumor cells. In the same way, DLBCL EVs carrying miR-125b-5p can reduce tumor sensitivity to rituximab by inhibiting TNFAIP3 expression and reducing CD20 expression (117). Whether the miR-125b-5p/TNFAIP3 axis can be used as a therapeutic approach for increasing DLBCL sensitivity to anti-CD20 antibodies requires further investigations.

EVs released by B cell could carry CD39 and CD73, two surface molecules known to hydrolyze ATP released by dying cancer cells into adenosine that hijacks CD8 T cell immune activity by binding the A2A adenosine receptors (118). One could speculate that B-cell-derived EVs may have a similar effect. The decrease of B-cell-derived EVs bearing CD73 and CD39 can be achieved by deregulating the docking protein RAB27A (118). This could be performed using an inactivated Epstein–Barr virus carrying siRNA, but it is also possible to generate EVs derived from cell lines producing RAB27A siRNA and to specifically deliver it to tumor cells.

Ultimately, thanks to their molecular structure mimicking the plasma membrane of the cells and their capability to reverse their cargo into target cells, exosomes could be shaped and filled of drug molecules, acting as drug-delivery systems. In fact, cancer vaccine clinical trials relying on the administration of exosomes produced by dendritic cells (Dexosomes), exploited to shuttle antigenic determinants of immune response, were conducted to immunize patients in the context of solid tumors (119–121). In the same way, systemic administrations of TNF-Related Apoptosis-Inducing Ligand (TRAIL)-armed exosomes have shown a great anti-tumor effectiveness against FL/DLBCL cell lines both *in vitro* and in a mouse model (122).

## Conclusion

Despite very interesting recent data highlighting BM as a survival niche for lymphoma B cells, numerous controversies remain open on the role of the BM versus LN niches during the early step of lymphomagenesis or at the stage of post-treatment minimal residual disease that could generate relapse. In FL, both pre-tumoral B cells and early committed precursor cells, that will give rise to overt FL, have been shown to be enriched in BM (22). However, transformation events required iterative passages throughout the GC making it difficult to define precisely whether BM is a primary or a secondary tumor niche. The influence of tumor genetics or patient features on the capacity of tumor B cells to home and develop into BM remains completely unexplored. A major limitation for all BM-dedicated studies is the limited availability of good quality samples to perform phenotypic, transcriptomic, and functional studies and the lack of iterative sampling allowing evaluation of the impact of disease evolution or therapeutic strategies. BM aspirates are scarce and do probably not include the whole diversity of tumor/TME components, in particular stromal cells. Moreover, fixed BM biopsies are very difficult to exploit for spatial transcriptomics and even multiplex immunohistofluorescence approaches. Such technical issue hampers a precise evaluation of spatial heterogeneity in B-cell lymphomas integrating BM as a key tumor site.

Altogether, many evidence support the clinical interest of targeting the crosstalk between BM stromal cells and malignant B cells, through the inhibition of stroma-derived B-cell growth factors, the mobilization of clonal B cells outside their supportive BM niche, or the reprogramming of tumor-supportive stromal cells. Identifying the best therapeutic options, and how to combine them with tumor-targeting drugs or immunotherapy approaches will be the major challenge in the field.

## Author Contributions

ED wrote the paper, SM reviewed the paper, and KT supervised and wrote the paper. All authors contributed to the article and approved the submitted version.

## Funding

This work was supported by research grants from Fondation ARC (PGA1 RF20170205386) and the Institut National du cancer (INCA AAP PNP19-009).

## Conflict of Interest

The authors declare that the research was conducted in the absence of any commercial or financial relationships that could be construed as a potential conflict of interest.

## Publisher’s Note

All claims expressed in this article are solely those of the authors and do not necessarily represent those of their affiliated organizations, or those of the publisher, the editors and the reviewers. Any product that may be evaluated in this article, or claim that may be made by its manufacturer, is not guaranteed or endorsed by the publisher.
